# Nordic Society of Paediatric Haematology and Oncology (NOPHO) Radiotherapy Working Group consensus guidelines on radiotherapy for paediatric low-grade gliomas

**DOI:** 10.2340/1651-226X.2026.44815

**Published:** 2026-02-15

**Authors:** Anna Embring, Tanja Skyttä, Jacob Engellau, Irina Kerna, Daiva Sendiuliene, Malin Blomstrand, Daniel J. Indelicato, Beate Timmerman, Yasmin Lassen-Ramshad, Henriette Magelssen

**Affiliations:** a Department of Oncology, Karolinska University Hospital, Stockholm, Sweden; b Department of Oncology-Pathology, Karolinska Institute, Stockholm, Sweden; c Department of Oncology, Tampere University Hospital, Tampere, Finland; d Department of Hematology, Oncology and Radiation Physics, Skane University Hospital, Lund, Sweden; e North Estonia Medical Centre, Tallinn, Estonia; f Department of External Beam Radiotherapy, National Cancer Institute, Vilnius, Lithuania; g Department of Oncology, Sahlgrenska University Hospital, Gothenburg, Sweden; h Department of Oncology, Institute of Clinical Sciences, University of Gothenburg, Gothenburg, Sweden; i Department of Radiation Oncology, University of Florida College of Medicine, Jacksonville, FL, USA; j West German Proton Therapy Centre Essen (WPE), Essen University Hospital, Essen, Germany; k Department of Particle Therapy, University Hospital Essen, Essen, Germany; l West German Cancer Centre (WTZ), German Cancer Consortium (DKTK), Essen, Germany; m Danish Centre for Particle Therapy, Aarhus University Hospital, Aarhus N, Denmark; n Department of Oncology, Oslo University Hospital, Oslo, Norway

**Keywords:** radiotherapy, paediatrics, brain neoplasms, practice guideline

## Abstract

**Background and purpose:**

Paediatric low-grade gliomas (pLGG) are the most common brain tumours in children. Radiotherapy, once the standard treatment for unresectable pLGG, is now used less frequently due to concerns about late side effects. The Nordic Society of Paediatric Haematology and Oncology (NOPHO) Radiotherapy Working Group aims to provide consensus guidelines on the use of radiotherapy for pLGG, addressing the current controversies and facilitating decision-making.

**Patient/material and methods:**

The guidelines were developed by clinical/radiation oncologists from the Nordic and Baltic countries and two international experts during a 2-day working group meeting. The meeting included presentations from the international experts and was preceded by a survey on radiotherapy practices and a non-systematic review of the literature on pLGG.

**Results:**

We present consensus-based recommendations for radiotherapy of pLGG. The guidelines discuss indications and timing of radiotherapy, age-related considerations, and the impact of genetic predisposition disorders such as neurofibromatosis type 1. Modern radiotherapy techniques, such as proton therapy, are highlighted for their potential to reduce long-term side effects.

**Interpretation:**

Radiotherapy remains the most effective treatment for unresectable pLGG, but its use must be carefully weighed against the risk of long-term side effects. The guidelines emphasise a personalised treatment approach, considering the evolving field of targeted therapies and the importance of multidisciplinary input in decision-making.

## Introduction

Paediatric low-grade gliomas (pLGG) are the most common brain tumours in children, making up approximately one third of all tumours in the central nervous system (CNS) [1]. According to the World Health Organization (WHO), pLGG are a heterogenous group of tumours in the brain or spinal cord classified as grade 1 or 2 tumours of the CNS [2]. Some of the more common types of pLGG are pilocytic astrocytoma, pilomyxoid astrocytoma, ganglioglioma, and diffuse astrocytoma. Recent molecular advances have revealed that with few exceptions, pLGG are driven by genetic abnormalities in the mitogen-activated protein kinase (MAPK) pathway, making targeted therapy a possible treatment option [3]. There are several studies showing promising results for these novel therapies, but there are still uncertainties regarding long-term effects [3].

Patients with pLGG have an excellent 10-year overall survival (OS) of over 90% [1], and surgery is the treatment of choice when resection can be performed without loss of function. Progression-free survival (PFS), however, varies considerably depending on the extent of surgery. According to a study done by the Children’s Oncology Group, which analysed 518 patients treated with primary surgery, the 8-year PFS was 93% for patients who underwent gross total resection and around 50% for those with residual tumours after surgery [4]. Radiotherapy was previously the standard treatment for unresectable pLGG, but is now reserved for selected cases due to concerns of late side effects such as neurocognitive decline, neurovascular events, and secondary tumours [5].

pLGG rarely transforms into high-grade gliomas, and may stop progressing and be quiescent when patient reach adulthood [6]. This is sometimes referred to as oncogene senescence [7]. Accordingly, glioma-related death is uncommon in adult pLGG survivors [8]. This behaviour makes pLGG different from low-grade gliomas in adults where malignant transformation is common and the 10-year survival is around 60% [9]. With the excellent OS seen in patients with pLGG, the disease is more and more considered being a chronic condition, where quality of life, preservation of function, and prevention of late effects must be taken into careful consideration when deciding on treatment modality. Asymptomatic pLGG does not always require treatment and patients are often followed by clinical observation and repeated magnetic resonance imaging (MRI). On the other hand, progressive or symptomatic pLGG will require treatment, which could be either surgery, radiotherapy, or systemic therapy in the form of targeted therapy or chemotherapy.

A Nordic Society of Paediatric Haematology and Oncology (NOPHO) Radiotherapy Working Group survey (described below) showed that in the Nordic countries, radiotherapy is rarely used in the treatment of pLGG today. Systemic treatment has become the therapy of choice when a tumour is not eligible for surgery [5]. This is somewhat in contrast to other countries, where radiotherapy is used more frequently in pLGG management. As an example, an international survey in 2016 showed that at the time, pLGG were the third most common paediatric brain tumour type treated with proton therapy [10]. European guidelines state that radiotherapy is a treatment option if minimal tumour growth imposes high risk of significant morbidity [5]. The main dilemma, however, is often that it is difficult to decide when this point is reached, as loss of function in pLGG patients is gradual over a long period of time, and it is challenging to define severe morbidity in a continuous decline. We believe that today some patients are offered radiotherapy too late, after multiple lines of systemic treatment and re-operation, and the result of the delay is loss of vital function and cumulative burden of iatrogenic toxicity. The aim of these NOPHO Radiotherapy Working Group guidelines is to complement existing European guidelines [5] and facilitate decision-making in when and how radiotherapy could be used in the management of pLGG.

## Material and methods

The NOPHO Radiotherapy Working Group consists of clinical/radiation oncologists from the Nordic and Baltic countries treating children with radiotherapy. These consensus guidelines are the result of a 2-day working group meeting on radiotherapy of pLGG. The meeting was attended by NOPHO Radiotherapy Working Group members from Denmark, Estonia, Finland, Lithuania, Norway, and Sweden. International experts of radiotherapy of pLGG were invited to present the European (B. Timmerman, Germany) and North American (D. Indelicato, US) perspective on the topic. The meeting was preceded by a survey on radiotherapy practice in pLGG sent out to the 33 members of the NOPHO Radiotherapy Working Group, and a non-systematic review of the literature on pLGG. After completing the guidelines, they were sent out to all members of the NOPHO Radiotherapy Working Group and the two international experts for review, and all agreed on the final version of the guidelines. The clinical practice guidelines are reported according to the AGREE reporting checklist [11].

## Results

### Indications and timing of radiotherapy

For decades, radiotherapy was the treatment of choice for progressive, unresectable pLGG due to treatment efficacy with studies showing 10-year OS and PFS of 96–98% and 68–80%, respectively [12, 13, 14], compared to chemotherapy showing a 5-year PFS of 34–53% [15]. Radiotherapy has a long-established place as the most effective non-surgical therapy. However, due to the risk of late side effects and the advent of new therapies, the use of radiotherapy has diminished [3]. The indication and timing of radiotherapy in pLGG is a matter of great controversy and is dependent on factors such as age of the patient, comorbidities, genetic pre-disposition disorders, tumour location, and resectability. The European Society for Paediatric Oncology (SIOPe) low-grade glioma working group state in their standard clinical practice recommendations that radiotherapy may be considered upfront for tumours that have a high risk of significant morbidity with minimal tumour growth or when the risk of radiotherapy-related neurocognitive side effects is low, provided that an agreement is reached at a multidisciplinary tumour board [5].

With the shift away from first-line radiotherapy, patients with tumour progression following chemotherapy are more common in radiation oncology clinics worldwide. This tumour progression can cause loss of function and necessitate increased target volumes. It is now evident that high-risk patients, with midbrain/hypothalamic tumour or unresectable diffuse astrocytoma, have inferior long-term survival and may benefit from early radiotherapy [6]. Furthermore, several studies showed improved visual acuity after radiotherapy of optic pathway glioma. In a phase II trial on conformal radiotherapy in pLGG patients, visual impairment decreased from approximately 30% before radiotherapy to <10% 12 months after radiotherapy [13].

### Age

In the study conducted by Merchant et al., patients younger than 5 years experienced a greater decline in cognition after conformal radiotherapy for pLGG compared to older patients [16]. Similarly, in a study of 32 patients treated for pLGG with protons, children < 7 years showed a significant decline in neurocognitive function after treatment [17]. Some studies suggest that older pLGG-patients (> 7–12 years) do not develop neurocognitive difficulties after treatment with radiotherapy [16, 17]. Younger age may also play a role in other late side effects. For example, age < 10 years has been shown to be associated with increased risk of vasculopathy [14]. Therefore, young age could be considered a relative contraindication for radiotherapy particularly in larger tumours. For some tumour locations, for example spinal cord or optic nerve, modern radiotherapy techniques can achieve target coverage without radiation dose to the brain and eliminate the subsequent risk of developing neurocognitive deficits or neurovasculopathy. Therefore, in very selected cases, younger children could be eligible for radiotherapy to prevent loss of function (e.g. vision) due to tumour growth.

### Neurofibromatosis type 1

Neurofibromatosis type 1 (NF1) is a tumour predisposition disorder with an increased risk of developing pLGG, particularly in the optic pathways. Around 20% of pLGG occur in children with NF1 [18]. Assessing long-term cognitive effects in pLGG patients, NF1-patients fare worse and often have preexisting cognitive difficulties before being diagnosed with pLGG [19]. Radiotherapy should generally be avoided when treating CNS tumours in NF1-patients as they have an increased risk of both developing secondary neoplasms [20] and vasculopathies [21]. However, it is worth noting that in the study done by Merchant et al., including 13 NF1-patients treated with conformal radiotherapy, none of them developed a secondary CNS-tumour during follow-up (median follow-up for total cohort was 89 months) [13]. In very selected cases, where treatment options are limited, radiotherapy for pLGG can still be considered also in NF1-patients when tumours threaten vital function.

### Target delineation

- GTV (gross tumour volume) should be delineated on MRI imaging (T2, T2 FLAIR and T1+Gd) [22].
- CTV (clinical target volume) margin is usually 5 mm (standard), but under certain circumstances (such as infiltrative tumour growth pattern), 10 mm can be considered for diffuse (grade 2) tumours [17, 22, 23]. In partially resected tumours, the CTV should also encompass the entire tumour bed.

### Dose prescription

There is no prospective randomised trial exploring the optimal radiation dose in pLGG. 45–54 Gy in 1.8 Gy fractions is commonly used, depending on age at treatment, extent of disease, and tumour localisation [5, 17, 22, 24]. In a subgroup analysis of patients with pilocytic astrocytoma treated in the HIT-LGG 1996 trial, doses > 50.4 Gy did not improve PFS [25], and similarly, in the retrospective study of Paulino et al. there was no significant difference in PFS in patients receiving ≤50,4 Gy or > 50.4 Gy [26]. In contrast, in the study by Indelicato et al., dose < 54 Gy was associated with inferior local control [23]. However, it is unclear if these results were influenced by the fact that patients with brainstem or spinal pLGG, locations with inherently worse prognosis, were more likely to be treated with doses < 54 Gy.

With available knowledge, it is reasonable to recommend 50.4–54 Gy in 1.8 Gy fractions to cranial pLGG and 50.4 Gy for pLGG of the spinal cord. If including the tumour bed after surgery results in a large volume (a volume that would risk resulting in unacceptable toxicity), one could consider treating the tumour bed to 45 Gy (1.5 Gy/fraction) and the GTV to 54 Gy (1.8 Gy/fraction) as a simultaneous integrated boost (SIB).

### Dose constraints

Table 1.

**Table 1:** Dose constraints.

Organ	Dose constraint	Outcome/aim	Ref.	Comment
Brainstem	D_max/0.1cc_ 56.6 Gy D_10%_ 55.4 Gy D_50%_ 52.4 Gy	Reduce risk of brainstem injury	[27]	In 1.8–2 Gy fractions
Spinal cord				
*Without* chemotherapy	Preferred: D_0.03 cc_ < 54 Gy D_1 cc_ < 50.4 Gy Acceptable: D_0.03 cc_ < 56 Gy D_1 cc_ < 54 Gy	Reduce risk of myelopathy	[28]	In 1.8–2 Gy fractions
*With* chemotherapy	Preferred: D_0.03 cc_ < 50.4 Gy D_1 cc_ < 45 Gy Acceptable: D_0.03 cc_ < 54 Gy D_1 cc_ < 50.4 Gy	Reduce risk of myelopathy	[28]	In 1.8–2 Gy fractions
Optic nerves and chiasm	D_max_ 55 Gy	< 5% risk of optic neuropathy	[29]	In 1.8–2 Gy fractions
Retina	Preferred: D_max_ 40 Gy Acceptable: D_max_ 50 Gy	< 5% risk of late retinopathy Reduce risk retinopathy	[29] [30]	
Temporal lobe	15_Gy_ < 20%	Reduce risk of neurocognitive decline	[17]	If possible, prioritise left temporal lobe
Hippocampus	V15_Gy_ < 20%	Reduce risk of neurocognitive decline	[17]	If possible, prioritise left hippocampus
Pituitary	Mean dose <40 Gy	Reduce risk of endocrinopathy	[17]	
	Mean dose < 21–22 Gy_EQD2_ to hypothalamic-pituitary axis	Estimated 20% risk of growth hormone deficiency and central hypothyroidism	[31]	
Hypothalamus	Mean dose < 40 Gy	Reduce risk of endocrinopathy	[17]	
	Mean dose < 21–22 Gy_EQD2_ to hypothalamic-pituitary axis	Estimated 20% risk of growth hormone deficiency and central hypothyroidism	[31]	
Cochlea	Mean dose < 35 Gy	< 5% risk of hearing loss	[32]	Without concurrent chemotherapy
Vascular structures	Preferred:D_max_ < 30 Gy to major cerebral arteries and Circle of Willis	At an attained age of 45 years the predicted incidence of stroke was 2.1–4.2% at 30 Gy	[33]	If important vascular structures (e.g. basilar artery or middle cerebral artery) are included in the CTV, dose reductions could be attempted to these structures.
	The ALARA-principle (As Low As Reasonably Achievable) should be applied	Even moderate doses (1–10 Gy) to neurovascular structures are associated with an increased risk of stroke	[34]	
Vertebrae	Avoid steep dose gradient in vertebrae before puberty	Reduce risk of spinal problems such as scoliosis	[35]	According to SIOPe radiotherapy working group guideline

### Spinal pLGG

Spinal pLGG is rare. The German LGG study group published a retrospective analysis of 128 paediatric patients with spinal pLGG and 12 of these patients had received radiotherapy. They concluded that a majority of all patients experienced disease progression, but that radiotherapy can achieve high and long-lasting local control and should therefore be considered as adjuvant treatment, especially for older children with localised disease [36]. In a smaller retrospective study, Brisson et al. found that proton therapy could offer long-term disease control with limited toxicity [37]. In this cohort of eight patients, the majority were treated with 45–50.4 Gy.

### Craniospinal irradiation

Craniospinal irradiation may be a treatment option in children with disseminated disease [22, 24, 38]. Treatment dose could be 36 Gy, in 1.8 Gy fractions, or in younger patients, 35.2 Gy in 1.6 Gy fractions, with a boost to 45–54 Gy to areas of gross disease.

### Treatment modality

In treatment of paediatric brain tumours, conformal radiotherapy is mandatory [39]. In the treatment of pLGG, proton therapy frequently offers a more favourable dose distribution compared to photon therapy and could be the modality of choice if accessible. In the meta-analysis by Kiss-Miki et al., paediatric brain tumour patients treated with protons scored better over a range of neurocognitive function tests including global IQ compared to patients treated with photon radiotherapy [40]. In the study done by Greenberger et al. investigating clinical outcome after proton treatment for pLGG, neurocognitive functions were relatively spared, which could advocate the use of proton beam radiotherapy when treating pLGG with radiotherapy [17]. In selected cases, for example no access to proton therapy, conventionally fractionated stereotactic photon radiotherapy (highly conformal radiotherapy) is a reasonable alternative option [41].

### Concurrent systemic treatment

Combining systemic treatment with radiotherapy offers no clear benefit in pLGG patients and may lead to increased toxicity. Therefore, it is usually avoided in pLGG treatment. To avoid the synergistic toxicity, a drug should be interrupted approximately five elimination half-lives before start of radiotherapy [42].

### Pseudo-progression

Pseudo-progression is defined as a transient tumour growth that stabilises or decreases with time [43]. The terminology to describe this phenomenon varies, and sometimes the terms pseudo-progression, radiation-induced contrast enhancement (RICE), and radionecrosis are used interchangeably. It is important to recognise pseudo-progression and differentiate the condition from true tumour progression to avoid unnecessary interventions. In children treated with radiotherapy for pLGG, pseudo-progression has been observed in 29–40% of patients. The median time to onset was approximately 6 months and persisted a median of 6.2–7.2 months [23, 43, 44]. In a retrospective study done by Tsang et al., over 25% of patients exhibited pseudo-progression more than 1 year post-radiotherapy. Moreover, the study indicated that pseudo-progression was more prevalent in patients treated for pilocytic astrocytoma (43%) and was correlated with improved event-free survival in this cohort [43].

Treatment of pseudo-progression is only indicated if the patient develops symptoms. Commonly used treatments are steroids and/or bevacizumab [45].

### Long-term side effects of radiotherapy for pLGG

Radiotherapy for paediatric brain tumours is associated with neurocognitive side effects dependent on age, radiation dose, treated volume, and concurrent chemotherapy [46]. Other important, and potentially life-threatening, late side effects after cranial irradiation are vasculopathies and secondary neoplasms. A German multi-centre study including 316 pLGG survivors showed that all pLGG survivors are at risk of long-term cognitive impairment, regardless of whether the patient underwent treatment or simply surveillance [19]. In the study conducted by Williams et al. analysing 29 patients treated for pLGG with photons in the time-period 1970–2004, 65% experienced grade ≥3 late side effects, with the most common being serious cognitive disability [47]. In this study, four patients (14%) died due to treatment complications, all occurring more than 10 years after completing treatment, and three patients (10%) died of secondary malignancies. However, in accordance with contemporary standards, most patients received 3D conformal photon therapy with a margin of 2–3 cm. This likely influenced the frequency and nature of observed side effects. In a SEER analysis including patients treated for pLGG between 1973 and 2015, the absolute risk of developing a secondary malignancy was 13% in the cohort treated with radiotherapy and 5% in the non-radiotherapy cohort [48]. In the previously mentioned study by Merchant et al., treating mainly with 3D conformal photon radiotherapy and 1 cm CTV margin, the cumulative incidence of vasculopathy was 4.8% at 7 years [13]. In a Childhood Cancer Survivor Study of 181 adult survivors of pLGG treated in earlier eras of 3D conformal photon therapy, the survivors treated with a combination of surgery and radiotherapy had lower estimated IQ, lower occupation scores, lower income, and less education than those treated with surgery alone [49]. In the PENTEC review on subsequent neoplasms it was estimated that a dose of 50 Gy to the brain would lead to an excess absolute risk of CNS malignancy of 3.9% at age 50 and 11.0% at age 75. The analysis also showed a significant dose-response relationship for developing subsequent neoplasms after CNS irradiation, which implies that improved dose conformality may reduce the risk of developing subsequent neoplasms [50].

## Discussion and conclusion

**Figure 1:**
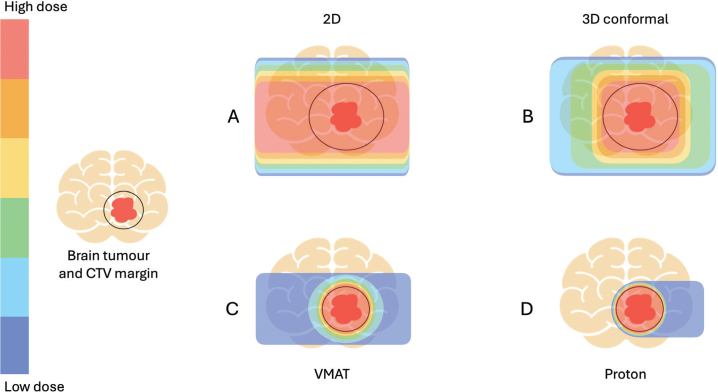
Schematic figures of radiation dose distributions of different treatment techniques. (A) 2D (2 dimensional) treatment with larger margins. Common treatment in the 1970s and 1980s. (B) 3D (3 dimensional) with intermediate margins. Common treatment of the 1990s and 2000s. (C) VMAT (Volumetric Modulated Arc Therapy). Contemporary, conformal photon treatment with smaller margin. (D) Contemporary, conformal proton treatment with smaller margin. CTV: clinical target volume.

Radiotherapy is the most effective treatment for unresectable pLGG but can be associated with severe long-term side effects. When considering radiotherapy as a treatment option, the risk of morbidity caused by tumour progression must be carefully weighed against the risk of late side effects from the treatment. The late side effects we see today, caused by radiotherapy given in the 70s–90s, are results of old treatment techniques (2D or 3D-conformal) most often using much larger margins (2–3 cm) than are standard today. It is reasonable to believe that radiotherapy delivered today, with conformal technique and smaller margins (often 5 mm), will result in less severe late side effects, as the radiation dose outside the tumour is largely reduced (Figure 1). However, this awaits confirmation, as the follow-up time for children treated with modern techniques remains limited and side effects may present decades post-treatment.

Management of pLGG requires a personalised treatment approach and – even taking the evolving field of targeted therapies into account – we believe that radiotherapy should be considered in selected cases of pLGG, including as a first line treatment in specific circumstances. For example, in unresectable tumours, impending loss of function (vision), or tumour progression on systemic treatment in sensitive areas, radiotherapy could be an effective treatment with acceptable risks of side effects. Unresectable tumours with high-risk features such as diffuse astrocytoma histology or midbrain/hypothalamic location might also benefit from early radiotherapy.

The study conducted by Bandopadhayay et al. is often referred to when advocating alternative treatment options to radiotherapy [8]. In this SEER analysis of 4040 patients with pLGG treated between 1973 and 2008, radiotherapy was associated with four times higher risk of dying of pLGG and 2.5-fold risk of dying of non-disease cause. However, assessing the survival curves carefully, the greatest difference in survival comparing survivors treated with or without radiotherapy occurs within the first 1–2 years of diagnosis. This by definition does not include second malignancies or other late effects of radiotherapy and instead implies a selection bias: Patients with poor prognosis were more likely to be treated with radiotherapy and this has skewed the results and conclusions of the study.

It is important to recognise that pLGG patients of an earlier era, such as those included in the SEER analysis, were treated with radiotherapy involving large CTV margins and less conformal treatment, contributing to common and severe late side effects [47]. Bandopadhayay et al. included patients treated over 50 years ago, but the perception endures today. Fortunately, modern studies have shown that CTV margins can be reduced substantially without compromising local control [17]. In addition, technical advances allow more conformal treatments, and early data suggest this evolution will result in less side effects and has prompted clinicians in the last 5 years to re-examine the role of radiotherapy in pLGG management [23].

**Figure 2:**
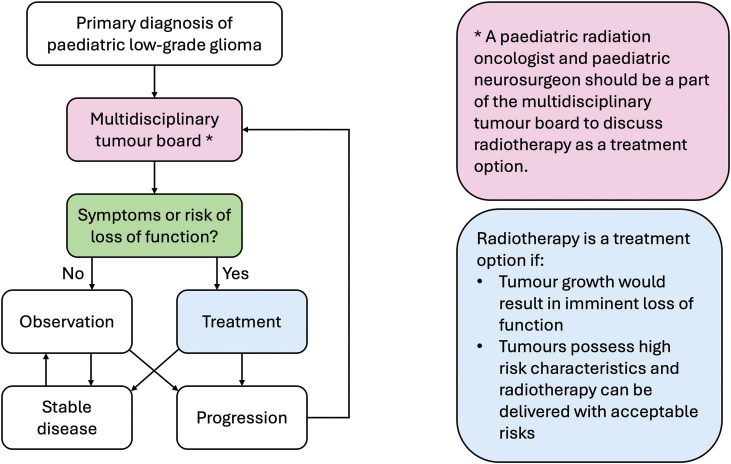
Proposed flow-chart for decision-making regarding treatment of paediatric low-grade glioma.

Like decision-making, communication with a family of a child with pLGG should be multi-disciplinary. Paediatric oncologists are best suited to discuss systemic therapy, neurosurgeons are best suited to discuss neurosurgery, and radiation oncologists are best suited to discuss radiotherapy. Therefore, radiation oncologists should actively participate throughout the disease trajectory, starting at diagnosis, to help families understand therapeutic options for pLGG (Figure 2). This could include meeting with patients and their families, to discuss pros and cons of different treatment options. Patient and family priorities are non-uniform: Some families prioritise disease control while others want to minimise side effects. Some prefer a shorter course of treatment, such as radiotherapy, to a longer period of systemic treatment that will inevitably tie the family to a hospital. This dialogue needs to continue into the survivorship period. For example, it is the radiation oncologist who knows the precise dose delivered to the Circle of Willis, and therefore might be best positioned to direct appropriate screening for vasculopathy [33].

These guidelines are compiled to facilitate decision-making in pLGG management and serve as a complement to existing pLGG treatment guidelines but have certain limitations. The absence of randomised trials comparing modern radiotherapy with chemotherapy or targeted therapy results in a lack of high-level evidence for the treatment of unresectable pLGG. Consequently, these guidelines are primarily based on consensus and expert opinion, with the inherent weaknesses of this method. Furthermore, our review of the literature not a systematic review. Another limitation is that with the rapid, both technical and medical, development of treatments, we do not have long-term results of the treatments we offer today and can only make assumptions of the outcome in the very long term.

In conclusion, pLGG management would benefit from multidisciplinary input when recommending the best treatment modality. Families would likewise benefit from multidisciplinary input when considering risks and benefits and selecting the treatment modality. With an excellent long-term efficacy, selected patients with unresectable disease can benefit from early radiotherapy to preserve function and prevent tumour morbidity.

## Supplementary Material



## Data Availability

Not applicable.
