# Supplementary material for: Nordic Society of Paediatric Haematology and Oncology (NOPHO) Radiotherapy Working Group consensus guidelines on radiotherapy for paediatric low-grade gliomas
Source: Acta Oncol. 2026 Feb 15;65:44815. doi: 10.2340/1651-226X.2026.44815 (PMC12926702; doi:10.2340/1651-226X.2026.44815)
Supplement: Supplementary file 1 [file AO-65-44815-s1.pdf]

Supplementary material has been published as submitted. It has not been copyedited, or typeset by Acta Oncologica

Survey sent out to all members of NOPHO Radiotherapy Working Group before the working group meeting and all answers submitted (blank boxes indicate no answer to the specific question from participating country).

Question	Denmark	Estonia	Finland	Lithuania	Norway	Sweden
In which situation do you give RT for pLGG in your institution/country?	Via MDT conference, but usually first when there are no more ”systemic therapy” options	Sometimes used if unresectable disease.	Rarely used.	If surgery or systemic treatment is not possible.	Via MDT. Mainly teenagers after multiple lines of systemic therapy.	Almost never (or never) in curative setting. Only clear indication is progressive spinal pLGG where chemotherapy is insufficient for local control
Is there a certain age limit, under which RT for pLGG would be considered as not indicated in your institution/country?	< 8 yrs, we might feel less comfortable by giving RT, if there are still systemic therapy options, as the LGG 2004 protocol had this as age limit in the RT group.			8-10 years	Depending on indication (and tumour location). Very reluctant <7-8 years	Depending on indication (and tumour location). Very reluctant <7 years.
Are there certain histological features, genetic vulnerabilities or	NF1			Li-Fraumeni, NF1	NF1, except on vital indication or as palliative treatment.	NF1, except on vital indication or as palliative treatment.

certain pLGG where RT would be considered as contraindicated in your institution/country?						
Would you treat without having a histological diagnosis?	Yes, for example, if it is an optical pathway glioma, with too much risk at the biopsy			Preferably not.	Preferably not, but sometimes (if biopsy has considerable risk)	Yes, under certain circumstances.
Definition of GTV: how do you define your GTV?	Residual Tumour			According to MRI images.	MRI	MRI (T2, T2 FLAIR and T1+Gd)
Do you include a ”tumourbed” (take earlier tumour extension into account)?	No			It depends on the size of postoperative bed, localisation.	Sometimes, depending on size, timing etc	Depending on volume and time since last surgery (if a very long time has passed, not as strong indication to include), but preferably if volume is not too large.
What CTV margin do you use?	3-5 mm			up to 1 cm	5 – (10) mm, tendency to smaller margins over the years,	5 (-10) mm

					so 5 mm nowadays	
What dose to you give to gliomas in the optic pathway region?	50.4 Gy			50,4 Gy	50.4 Gy, 1.8 gy/fraction	45-50.4 Gy in 1.8 Gy fractions
What dose do you give to gliomas in the fossa posterior?	50.4 Gy			45-54 Gy	50.4 – 54 Gy, 1.8 Gy/fraction	45-50.4 Gy in 1.8 Gy fractions
What dose do you give to gliomas in other locations?	50.4 Gy			45-54 Gy	50.4 – 54 Gy, 1.8 Gy/fraction	45-50.4 Gy in 1.8 Gy fractions, (higher if poor prognostic factors, as mixed grade 2-3)
Do you treat with protons or photons, if it is different from patient to patient what are your criteria to choose the modality?	Protons per default if <18 years	No access to protons. Conformal photon treatment is more commonly used.		We do not have access to protons, but I think pLGG would be an indication.	Protons or comparative dose planning protons/photons	Comparative dose planning protons/photons
Do you ever use SRS/gamma knife, LITT or ablative treatment for low grade gliomas?	No			No	Yes	Gamma Knife

Do you allow concomitant systemic treatment? If yes, which drugs?	Only Bevacizumab			In consultation with paediatric oncologist.	Preferably not	Preferably not
If no, what time interval do you usually want to have between end of systemic therapy and start of RT?	At least 4 weeks.			In consultation with paediatric oncologist.	Depending on half-time of drug	Depends on half-time of drug used.

RT – radiotherapy, pLGG – paediatric low-grade glioma, NF1 – neurofibromatosis type 1
